# US law enforcement policy predictors of race-specific police fatalities during 2015–16

**DOI:** 10.1371/journal.pone.0252749

**Published:** 2021-06-23

**Authors:** Marilyn D. Thomas, Alexis N. Reeves, Nicholas P. Jewell, Eli K. Michaels, Amani M. Allen

**Affiliations:** 1 Department of Epidemiology and Biostatistics, School of Medicine, University of California, San Francisco, San Francisco, CA, United States of America; 2 Department of Psychiatry, School of Medicine, University of California, San Francisco, San Francisco, CA, United States of America; 3 Department of Epidemiologic Science, University of Michigan School of Public Health, Ann Arbor, MI, United States of America; 4 Division of Epidemiology, School of Public Health, University of California, Berkeley, Berkeley, CA, United States of America; University of Arkansas for Medical Sciences, UNITED STATES

## Abstract

Mounting evidence suggests that law enforcement organizational factors contribute to higher incidence and racial disparities in police killings. To determine whether agency policies contribute to race-specific civilian fatalities, this exploratory study compared fatality rates among agencies with and without selected policies expected to reduce killings. A cross-section of 1085 fatalities in the 2015–2016 The Counted public-use database were matched to 481 agencies in the 2013 Law Enforcement Management and Administrative Statistics (LEMAS) database. Negative binomial regression estimated incidence rate ratios (IRR) adjusted for agency type, number of officers, percent female personnel, median income, percent with a bachelor’s degree, violent crime rate, and population size, with inference using robust standard errors. Agencies with greater proportions of full-time personnel (range 43–100%) had lower rates of all (IRR = 0.85; 95% confidence interval [CI] = 0.77–0.93) and non-White civilian killings (IRR = 0.85; CI = 0.73–0.99). Mission statements predicted lower rates of all (IRR = 0.70; CI = 0.58–0.84) and White killings (IRR = 0.60; CI = 0.40–0.90). Community evaluation and more types of personnel incentives predicted lower rates of White (IRR = 0.82; CI = 0.68–0.99) and non-White killings (IRR = 0.94; CI = 0.89–1.00), respectively. Increasing video use predicted higher rates of White killings (IRR = 1.13; CI = 1.01–1.28). No policies were significantly associated with Black civilian killings. Law enforcement policies that help reduce police killings may vary across racial groups with the least benefit for Black civilians. Impact evaluations and meta-analyses of initiatives aimed to mitigate fatalities should be explored, particularly policies to address anti-Black bias. A national registry tracking all police killings and agency policies is urgently needed to inform law enforcement policies aimed to mitigate civilian fatalities.

## Introduction

Public concern over recent police killings (i.e., police-caused deaths [1]), particularly among Black civilians, has inspired epidemiologic investigations to identify key drivers of racial disparities in police use of deadly force [2–13], or fatal *legal intervention*. The government defines legal intervention as “injuries inflicted by the police or other law-enforcing agents, including military on duty, in the course of arresting or attempting to arrest lawbreakers, suppressing disturbances, maintaining order, and other legal action” [14]. Studies have identified four central determinants of fatal legal intervention: individual officer and civilian characteristics, situational factors during encounters, neighborhood context, and organizational factors [15–29]. Research implicates racial bias against Black civilians as a potential determinant of racial inequities in police use of lethal force [5, 8, 11, 12, 30–39]. Much of the literature on reducing police fatalities has focused on officer and/or civilian behavior during encounters [18, 20, 22, 26, 40–44], community dynamics [13, 17, 18, 28], and police culture [45–47]. Organizational policies bi-directionally influence police culture (e.g., routine internal officer evaluation and high standards for officer conduct) which, in turn, shapes officer behavior [48–50]. Hence, organizational policies—concise regulations, procedures, and best practices that set the ethical and moral standard of operations and employee conduct within law enforcement agencies—may influence the incidence of police killings yet has received less attention in the epidemiologic literature.

Internal rules and regulations are affected by the underlying institutional attitudes permeating a law enforcement agency [49, 50]. Rather than solely holding individual officers accountable for preventable events, researchers have evaluated differences in the policies employed within agencies, and whether and to what extent those differences contribute to avoidable fatalities (e.g., police shootings) [50–55], assuming that some are unavoidable (e.g., police vehicle accidents). Though scant findings are mixed, the evidence points to several policies that may play a role including officer and agency oversight, collective bargaining, officer field training, minimum educational requirements, video use, and less lethal weapons use [17, 20, 21, 27, 56–61].

Despite modest evidence that organizational policies and programs may effectively reduce deadly use of force, there are substantial limitations to the generalizability and comparability of study findings. Because of limited data availability, many investigations and interventions focus on one or a few large cities or agencies of interest to examine the impact of differing agency characteristics [25, 42, 50, 51, 62–67]. Moreover, the wide variation in denominators used to estimate population rates of police killings (e.g., residents, police encounters, arrests) make study results lack comparability and national studies are scarce. Most studies using national administrative policies to estimate associations with police killings restrict the sample to large municipal agencies [13, 17, 24, 57, 58, 68]. However, public concern of unjust and racial inequities in police killings has been reported in both urban and rural US communities in recent years. Given this growing national crisis, evidence is needed among varying jurisdiction sizes to identify wide-ranging agency policies that impact police fatal use of force nationally.

The objective of this exploratory study was to compare police-caused fatality rates during 2015–2016 among United States (US) law enforcement agencies with and without selected policies. All agencies were analyzed, and then separate analyses of agencies only involved in White, Black, and non-White civilian killings were evaluated to assess potential differences across racial groups. Because use of force policies have been linked to lower police killings for Black but not White civilians [53], associations were hypothesized to be the most protective for agencies involved in Black (vs. White or non-White) civilian killings.

## Materials and methods

### Study sample

Data are from The Counted and the Law Enforcement Management and Administrative Statistics (LEMAS) databases [69, 70]. The Counted, compiled by *The Guardian* newspaper, is a public-use database of the total number of people killed by US law enforcement during 2015–2016 (*N = 2*,*238*). The Counted database was selected because it includes all police agencies involved in each civilian death as well as each decedent’s age, race, gender, armed status, and location and cause of death. Reporters validate data through open source reporting, police records, witness accounts, and family/friends. The database is promptly maintained and updated by *The Guardian* reporters and interactive journalists by collecting tips from the public, crowdsourcing, and data from the Federal Bureau of Investigation. Like many crowdsourced databases of police killings [71, 72], *The Guardian’s* database has been shown to have greater validity and reliability than government databases due to government agencies underreporting or misclassifying police-involved deaths [71, 73, 74].

LEMAS is a widely-used cross-section of information collected approximately every three years from a nationally representative sample of US law enforcement agencies regarding organizational characteristics (e.g., administrative policy, personnel characteristics, officer requirements) [13, 17, 18, 24, 57, 58, 75]. To ensure policies were implemented prior to each killing, the 2013 LEMAS database was used to match 2,826 agencies to 2015–2016 fatalities in The Counted according to the agency name(s) reportedly involved and the state in which the killing occurred. Because the unit of analysis was each agency involved, multiple agencies could be linked to any given fatality. The matching procedure resulted in a final sample of 481 agencies (17% of LEMAS) involved in 1,085 civilian deaths (49% of The Counted). Bivariate analyses of the matched and unmatched groups were assessed for variation in civilian characteristics (S1 Table) and policy implementation (S2 Table). We exclude the small number of state agencies (*N = 30*) because (1) state agencies and jurisdictions are very different than municipal and county agencies in demographic factors and their primary role in enforcing the law within their entire jurisdiction (e.g., state agencies are the main source of law enforcement in very small proportions of their jurisdictions) and (2) state agencies have different policies, training, and resources that may bias associations with fatality rates (e.g., community evaluation is less likely to exist within state agencies). This method resulted in 323 municipal agencies and 128 county agencies for analysis (*N = 451*).

### Exposure assessment

Thirteen LEMAS-reported policies expected to reduce lethal intervention were evaluated as exposures. Each policy aims to enhance organizational and officer performance or indirectly promote performance. Therefore, we expected each policy to help reduce avoidable killings and hypothesized lower predicted fatality rates in agencies *with* versus *without* these policies, especially initiatives specifically targeting reductions in officer use of deadly force and racial disparities (e.g., less-lethal weapons use, community policing). Data were coded such that a higher value indicates that the policy was reportedly implemented. Based on prior empirical and theoretical evidence, we evaluated policies related to agency climate and accountability, officer requirements, and personnel incentives that may mechanistically influence police killings. Each policy is described in more detail below: the proportion of full-time personnel [17], union representation (offered and active) [17], an established mission statement [8, 65], minimum educational requirements [20, 21, 42], additional training for new hires [11, 22], community policing training [57], less-lethal weapon use [62], video use [59, 76, 77], evaluation of citizen satisfaction with police [78–80], incentive compensation [21, 81, 82], and statistical analysis or research (any and external) [22].

The following variables were assessed dichotomously (No = 0; Yes = 1): *Collective bargaining* ‘*offered’* among sworn personnel; amid the subset of those that offered bargaining, *‘active’ collective bargaining*; having an established *mission statement*; at least 8 hours of training *community policing training* offered; *additional training* requirements for new-hires; *community evaluation* of resident satisfaction with agencies; agency accountability as *any statistical analysis* or research conducted; among the subsample reporting analysis, *external statistical analysis* (i.e., employing an organization outside of the agency); and *some college required* for new-hires.

The remaining policies were assessed continuously. The proportion of *full-time sworn personnel* (i.e., 0–100%) was measured among those full-time and part-time sworn and non-sworn. Summary scores were generated for less-lethal weapon use, video use, and incentive compensation. Offering personnel less-lethal weapon authorization was coded dichotomously across 9 types of weapons or actions (e.g., soft projectiles, neck restraints) with a summary score (range 0–9) representing more types of *less-lethal weapons* offered. Four video camera types (e.g., body, vehicle) were coded dichotomously: due to small cell sizes, the top two levels were combined resulting in a summary score (range 0 to 3) reflecting increasing *video use* types. Incentives included nine domains related to increased salary or additional paid leave (e.g., bi/multi-lingual, merit/performance), were coded dichotomously, and then summed (range 0–9) to represent increasing *incentives* offered.

### Outcome assessment

Although measures of police–civilian encounters and civilian arrests have been used to evaluate racial disparities in police use of force, population-level measures are likely the most robust [10, 16, 23, 38, 83–86]. This is because police encounters are systemically biased against minorities [23, 38, 83–85]. A countervailing opinion is that not every member of the population is at risk of being a police fatality victim [38, 84, 85]. In fact, all Black civilians are at risk of a police encounter even while doing acts that would not subject Whites to the same risk (e.g. jogging in a predominantly White neighborhood). Therefore, conditioning estimates on police–civilian encounters or subsequent arrests using administrative records can produce biased results. This type of statistical bias can lead to underestimation of racial inequities: non-White rates have inflated denominators (more encounters) while White rates have deflated denominators (fewer encounters) thereby making racial groups more statistically similar [84]. Hence, incidence (i.e., fatality) rates were calculated using the total number of killings within an agency (numerator) divided by the total jurisdiction population size (denominator). Because racial inequities in police use of lethal force point to Black civilians being at the greatest risk [5, 12, 35, 39, 87, 88], agency policies found to be protective for Black civilians may benefit all minoritized racial groups. Therefore, we conducted separate analyses for agencies involved in all, White, Black, and non-White civilian killings. Race-specific rates were estimated using race-specific population size to avoid bias from conditioning on denominators that may over- or under-represent racial groups within a given jurisdiction. Apart from “Hispanics”, racial categories were non-Hispanic. The search tool Social Explorer was used to collect demographic data from the 2016 American Community Survey to measure the total and race-specific population distributions within a given jurisdiction [89].

### Covariates

Empirically established and theoretical confounders were identified using a directed acyclic graph (DAG) (S1 Fig); civilian race and agency and jurisdiction-specific characteristics [17, 20, 21, 28, 57, 58, 68, 90]. *Race* categories included White, Black, and non-White (Black, Asian/Pacific Islander, Hispanic, Native American, Arab-American, Other/Unknown). *Agency type* included municipal and county agencies. Using LEMAS-established cut-points, *number of officers* was measured dichotomously (<100, ≥100). Consistent with the US Census Bureau [91], *U*.*S*. *region* was categorized as Midwest, Northeast, South, and West. All remaining covariates were evaluated continuously. Although the link between agency diversity and police killings is not well-established [68, 90], use of force has been shown to vary by officer race and gender [20, 92–94] suggesting that agency demographic composition may have implications for policy implementation; hence, we controlled for both racial and gender diversity. Gender diversity measured the number of *female sworn personnel* among all sworn personnel. Following previous work, racial diversity was measured as the proportion of *full-time “White” sworn personnel* [57]. Data from Social Explorer [89] measured percent *native*, *educational attainment (≥HS & ≥BA/BS)*, *poverty rate*, *owner occupied housing units*, *and married*, as well as *Gini index* (perfect income equality to perfect income inequality [range 0–1]), whereas the median was used for a*ge* and *income*. The Uniform Crime Report measured *violent crime rate*—the average crimes reported per 100,000 people [95].

### Statistical analysis

Analysis was restricted to agencies involved in one or more civilian deaths during 2015–2016. Missingness was negligible (3%), nonetheless we performed multiple imputation (MI) (*m* = 10) [96]: the MI assumption of data missing at random, although ultimately unverifiable, was supported by the similarity of MI and complete case results. Eighteen civilian deaths were dropped due to mismatches between agency name and state. One tribal agency with one Native American fatality was omitted due to inaccessible information on jurisdiction characteristics. One municipal agency outlier (i.e., exceptionally large jurisdiction size) was excluded. The final sample was comprised of 449 municipal and county agencies involved in at least one civilian killing during 2015–2016. Descriptive statistics reported for all agencies include all races. Because multiple agencies can be involved in one civilian killing, race-specific sample sizes will not sum to equal that of all agencies. Bivariate analyses (e.g., χ^*2*^) evaluated associations between covariates and each agency policy and each fatality group.

Model specification involved theory-based (e.g., DAGs) and data-driven approaches to assess confounding, collinearity, and best model fit. To address over-dispersion of the count outcome variable, negative binomial regression using robust standard errors estimated incidence rate ratios, adjusted for covariates either significant with two-sided *P*<0.05 or theoretical (i.e., “forced-in” regardless of statistical significance). The *a priori* minimum sample size required was calculated as 388 agencies (15 potential predictors, 1-β = 0.80, α = 0.05, and *f*^2^ = 0.05) [97]; p-values were reported at *P*<0.10 to capture marginally significant estimates within the smaller race-specific sample sizes. Each policy was examined in a separate adjusted model. Sensitivity analyses involved adjusted models restricted to agencies with jurisdiction sizes of 35,000 people or more because this was about the minimum-sized county population whereas the maximum-size of municipal and county populations were similar. STATA 16 was used for all statistical analyses.

## Results

### Sample characteristics

Table 1 shows the sample distribution of agency policies by those involved in all, White, Black, and non-White civilian killings. Table 2 shows the sample distribution of covariates. Overall, agencies involved in civilian killings were largely municipal (72%), had 100 or more officers (72%), were located in the South (46%), and had high Black population densities compared to the general population (46% vs. 13%). Full-time sworn personnel were mostly White (75%) and male (90%). Bivariate analyses are presented in S3 Table.

**Table 1:** Distribution of 2013 LEMAS policies among agencies involved in all and race-specific police killings in The Counted during 2015–2016.

	All Agencies w/Killings	Agencies w/White Killings	Agencies w/Black Killings	Agencies w/non-White Killings
Policy	*(N = 449)*	*(n = 297)*	*(n = 165)*	*(n = 254)*
	*n (column %)*			
% Full-time personnel				
*mean (±SD)*	94.9 (7.5)	94.7 (8.4)	95.2 (5.8)	95.6 (5.7)
Collective bargaining offered				
No	71 (16)	50 (17)	21 (13)	30 (12)
Yes	378 (84)	247 (83)	144 (87)	224 (88)
^Ɨ^Active collective bargaining				
No	160 (43)	100 (41)	65 (45)	88 (39)
Yes	214 (57)	144 (59)	79 (55)	135 (61)
Mission statement				
No	15 (3)	11 (4)	3 (2)	5 (2)
Yes	433 (97)	434 (96)	162 (98)	249 (98)
Some college required				
No	377 (84)	244 (82)	132 (80)	206 (81)
Yes	72 (16)	72 (18)	33 (20)	48 (19)
Additional training for new hires				
No	39 (7)	24 (8)	24 (5)	16 (6)
Yes	410 (93)	273 (92)	156 (95)	238 (94)
Community policing training				
No	151 (34)	103 (35)	59 (36)	85 (33)
Yes	298 (66)	194 (65)	106 (64)	169 (67)
Less-lethal weapons				
*range 0–9; mean (±SD)*	7.2 (1.5)	7.2 (1.6)	7.1 (1.5)	7.3 (1.5)
Video use				
*range 0–3; mean (±SD)*	1.4 (0.9)	1.4 (0.9)	1.4 (0.9)	1.4 (0.9)
Community evaluation				
No	249 (55)	164 (55)	77 (47)	131 (52)
Yes	200 (45)	133 (45)	88 (53)	123 (48)
Incentives				
*range 0–9; mean (±SD)*	3.1 (1.9)	3.1 (1.9)	3.3 (1.8)	3.5 (1.8)
Any statistical analysis				
No	36 (8)	27 (9)	6 (4)	11 (4)
Yes	413 (92)	270 (91)	159 (96)	243 (96)
^£^External statistical analysis				
No	312 (76)	203 (76)	110 (70)	178 (74)
Yes	97 (24)	65 (24)	48 (30)	62 (26)

Abbreviation: SD = standard deviation.

Note: All agencies’ estimates include all races hence Black and White agency estimates will not sum to equal all agencies.

^Ɨ^All agencies (n = 374); Agencies w/White killings (n = 244); Agencies w/Black killings (n = 144); Agencies w/non-White killings (n = 223).

^£^All agencies (n = 409); Agencies w/White killings (n = 268); Agencies w/Black killings (n = 158); Agencies w/non-White killings (n = 240).

**Table 2:** Distribution of organization and jurisdiction characteristics among agencies involved in all and race-specific police killings in The Counted during 2015–2016.

	All Agencies w/Killings	Agencies w/ White Killings	Agencies w/ Black Killings	Agencies w/non-White Killings
*Organization Characteristics*	*(N = 449)*	*(n = 297)*	*(n = 165)*	*(n = 254)*
Agency type	*n (column %)*			
County	127 (28)	94 (32)	36 (22)	54 (21)
Municipal	322 (72)	203 (68)	129 (78)	200 (79)
US region				
West	143 (32)	95 (32)	34 (21)	90 (35)
Midwest	71 (16)	53 (18)	24 (15)	29 (11)
South	208 (46)	135 (45)	95 (58)	119 (47)
Northeast	27 (6)	14 (5)	12 (7)	16 (6)
# Officers				
<100	125 (28)	88 (30)	16 (10)	42 (17)
100+	324 (72)	209 (70)	149 (90)	212 (83)
	*mean (±SD)*			
% Female personnel	10.1 (5.5)	11.0 (5.1)	13.2 (5.8)	11.9 (5.7)
% White personnel	75.1 (2.4)	78.6 (2.4)	69.7 (2.2)	68.1 (2.4)
***Jurisdiction Characteristics***				
Population size (100K)	289.2 (489.3)	313.1 (538.8)	440.3 (637.9)	381.4 (572.9)
% Black population	45.6 (98.1)	42.5 (102.6)	90.8 (138.8)	66.6 (119.3)
Median age	36.7 (5.2)	37.2 (5.5)	36.2 (4.7)	36.7 (4.6)
% US native	86.7 (11.0)	87.9 (10.2)	85.2 (11.0)	84.1 (11.4)
% HS Diploma	85.9 (7.7)	87.1 (6.1)	85.5 (7.1)	84.4 (8.6)
% BA/BS Degree	28.4 (11.8)	29.1 (11.6)	29.9 (11.4)	28.5 (11.6)
Gini index	0.47 (0.22)	0.47 (0.27)	0.50 (0.35)	0.48 (0.29)
Median income (K)	53.9 (17.1)	55.0 (16.9)	50.9 (15.2)	52.4 (15.4)
% Poverty	16.8 (7.1)	15.8 (6.4)	18.9 (7.5)	18.2 (7.3)
% Married	46.9 (7.9)	48.2 (7.4)	42.9 (7.7)	44.7 (7.6)
% Owner occupied housing	57.7 (12.0)	59.5 (11.6)	52.8 (11.7)	54.3 (11.4)
Violent crime rate (100K)	409.5 (364.8)	356.0 (318.8)	584.2 (426.1)	517.5 (389.8)

Abbreviations: HS = high school; BA/BS = bachelor’s; SD = standard deviation; K = 1,000.

### Regression model specification

Number of officers, percent female personnel, percent with a HS diploma or bachelor’s degree, median income, percent poverty status, violent crime rate, percent Black population, and total population size contributed to 99.3% of the principal components (eigenvalues>1). Among these, the highly correlated socioeconomic and demographic variables (*P*<0.05) contributing the least to the variance were excluded; percent with a HS diploma, poverty, and total population size. Bivariable analysis showed that each remaining covariate confounded the exposure–outcome association independently (*P*<0.05). Nested modeling and Akaike and Bayesian information criteria indicated that violent crime rate did not improve model fit; however, it was considered an important theoretical confounder and thereby was included in the analysis.

### Multivariable negative binomial regression

Table 3 shows adjusted incidence rate ratios (IRRs) comparing rates of police killings between US law enforcement agencies with and without selected policies. All models were adjusted for agency type, number of officers, percent female personnel, median income, percent with a bachelor’s degree, and violent crime rate. To account for variation in jurisdiction size, race-specific models were further adjusted for total population size. Given that total population size was used to calculate the incidence rate for all civilian killings, models including all agencies were further adjusted for Black population density to account for size differences in the subpopulation at highest risk of being killed by police [2, 3, 7, 12].

**Table 3:** Multivariable negative binomial regression of municipal and county-level police killing rates on 2013 LEMAS policies by agencies in The Counted during 2015–2016, counts by all and race-specific killings.

	^a^All Agencies w/Killings *(N = 449)*			^b^Agencies w/White Killings *(n = 297)*			^b^Agencies w/Black Killings *(n = 165)*			^b^Agencies w/Non-White Killings *(n = 254)*		
Policy	IRR	LB 95% CI	UB 95% CI	IRR	LB 95% CI	UB 95% CI	IRR	LB 95% CI	UB 95% CI	IRR	LB 95% CI	UB 95% CI
% Full-time personnel	*0.847	**0.771**	**0.930**	0.966	0.861	1.084	1.975	0.765	1.243	**0.850**	**0.732**	**0.987**
Collective bargaining	0.958	0.778	1.180	0.938	0.771	1.142	0.838	0.524	1.339	0.919	0.601	1.404
^Ɨ^Active bargaining	0.977	0.836	1.142	1.103	0.903	1.348	1.128	0.833	1.527	0.924	0.754	1.133
Mission statement	*0.696	**0.576**	**0.841**	**0.595**	**0.395**	**0.897**	0.959	0.658	1.397	0.681	0.404	1.149
Some college required	1.121	0.963	1.304	1.017	0.790	1.309	1.139	0.833	1.557	0.870	0.695	1.088
Additional training	*1*.*274*	*0*.*973*	*1*.*668*	1.041	0.831	1.305	0.908	0.560	1.472	1.207	0.802	1.816
Community policing	0.967	0.843	1.108	0.975	0.823	1.156	0.890	0.666	1.191	0.987	0.812	0.200
Less-lethal weapons	0.991	0.944	1.039	0.972	0.917	1.029	*1*.*091*	*0*.*998*	*1*.*192*	1.006	0.939	1.078
Video use	1.020	0.945	1.102	**1.133**	**1.005**	**1.278**	0.940	0.815	1.083	1.018	0.904	1.146
Community evaluation	0.919	0.801	1.053	**0.823**	**0.683**	**0.992**	1.052	0.816	1.356	1.071	0.873	1.314
Incentives	0.986	0.948	1.025	0.997	0.941	1.056	1.041	0.954	1.137	**0.941**	**0.885**	**0.999**
Any statistical analysis	0.884	0.652	1.198	1.005	0.745	1.355	0.706	0.279	1.787	0.735	0.362	1.494
^£^External analysis	1.015	0.870	1.184	0.983	0.776	1.245	*0*.*799*	*0*.*619*	*1*.*031*	0.983	0.822	1.175

Abbreviations: LEMAS = Law Enforcement Management and Administrative Statistics; IRR = incidence rate ratio; CI = confident interval; LB = lower bound; UB = upper bound; HS = high school.

^a^Estimates adjusted for agency type, number of officers, percent female personnel, median income, percent with a Bachelor’s degree, violent crime rate, and percent Black population.

^b^Estimates adjusted for agency type, number of officers, percent female personnel, median income, percent with a Bachelor’s degree, violent crime rate, and jurisdiction population size.

^Ɨ^All agencies (n = 374); Agencies w/White killings (n = 244); Agencies w/Black killings (n = 144); Agencies w/non-White killings (n = 223).

^£^All agencies (n = 409); Agencies w/White killings (n = 268); Agencies w/Black killings (n = 158); Agencies w/non-Whites Killings (n = 240).

**Bolded** =

*P<0.01, P<0.05; Italicized = P<0.10.

Higher proportions of full-time sworn personnel were associated with lower fatality rates for agencies involved in all (IRR = 0.85; 95% confidence interval [CI] = 0.77–0.93) and non-White civilian killings (IRR = 0.85; CI = 0.73–0.99). Having a mission statement predicted lower rates of all (IRR = 0.70; CI = 0.58–0.84) and White killings (IRR = 0.60; CI = 0.40–0.90). Offering personnel incentives predicted a lower non-White rate (IRR = 0.94; CI = 0.89–1.00). Agencies participating in community evaluation had a lower White rate (IRR = 0.82; CI = 0.68–0.99). Greater video use predicted a higher White rate (IRR = 1.13; CI = 1.01–1.28). Additional training for new-hires predicted a marginally higher rate of all killings (IRR = 1.27; CI = 0.97–1.67). Greater use of less-lethal weapons predicted a marginally higher Black rate (IRR = 1.09; CI = 1.00–1.19). Utilizing an external organization to conduct internal statistical analysis or research had a marginally lower Black rate (IRR = 0.80; CI = 0.62–1.03). Our sensitivity analyses showed that restricting the sample to jurisdiction sizes of 35,000+ people did not meaningfully change the magnitude or direction of any estimates, though, unsurprisingly, precision was reduced or remained unchanged.

## Discussion

We leveraged national data to provide evidence of the role that US law enforcement policies may play on police killings of civilians by racial group. This observational study was not intended to establish causation. Moreover, the lack of complete LEMAS data to match to all killings in The Counted render our findings exploratory; results should be interpreted with caution. That said, our results are strongly suggestive of strategies worth evaluating among agencies involved in civilian fatalities. As hypothesized, nearly all policies were protective. Contrary to our hypothesis, (1) associations were stronger for agencies involved in White (vs. Black) killings, (2) one policy was harmful for White rates, (3) policies intended to combat avoidable fatalities in racially minoritized communities were statistically null for Black and non-White rates but not White rates, and (4), most notably, no policies were significantly associated with a change in Black killing rates. Prior evidence has shown officer-level interventions to be ineffective at reducing use of force [58, 76, 77, 98] or mitigating racial disparities [88]. Our results show that agency-level policies related to organizational culpability may be effective, albeit differentially by racial group. Our findings suggest that White civilians may benefit more from agency policies that help mitigate avoidable police killings than civilians of color.

### Full-time sworn personnel

Our finding that agencies with more full-time personnel had lower rates of all and non-White civilian killings is novel. One study reported that agencies with more full-time sworn officers had lower citizen complaints while those having internal affairs units with full-time personnel had higher complaint rates, which the researchers suggest could represent the presence of more structured internal policies and procedures [17]. We found null associations for White and Black civilian subgroups, suggesting a greater influence of full-time personnel on more racially diverse communities. Our findings support the commonly cited hypothesis that a more structured police organization may lead to greater officer accountability and improved officer behavior [17, 49, 58]. The mechanism linking full-time personnel and police killings through organizational structure is unclear and warrants further consideration.

### Mission statement

Having an established mission statement predicted lower rates of all and White civilian killings, which has not yet been reported. Police agency mission/value statements communicate the fundamental beliefs that govern organizational policy, priorities, and commitment to the community served. The null findings for agencies involving racially minoritized groups (vs. White) was surprising given they were slightly more likely to have a mission statement (98% vs. 96% respectively). Future qualitative content analysis of language used in established mission statements (e.g., diversity or equal protection component) as predictors of officer use of deadly force might help elucidate its role.

### Community evaluation

Inconsistent with related work, community evaluation was protective in this study, but only for White civilian killings. Agencies utilizing citizen/community review boards have reported increased excessive use of force complaints yet study findings are not disaggregated by civilian race [17, 99]. In the current study, agencies involved in White fatalities had the lowest proportion of community evaluation. Our findings suggest that White civilians may benefit from community evaluation initiatives, more so than other racial groups. Further, jurisdictional differences in the sociodemographic composition may influence whether community evaluation is implemented and to what extent it benefits minoritized populations.

### Incentives

We are seemingly the first to report increased personnel incentives associated with lower non-White civilian fatalities. Seminal works by Uelmen (1973) and Sherman (1983) posited that changes to organizational incentives may mitigate avoidable police killings [50, 54]. Similarly, McElvain and Kposowa (2008) recommended officer college education incentives after finding that college-educated (vs. no college) officers were less likely to be involved in a shooting [20]. However, current research on organizational regulations aimed to reduce police killings largely focus on restrictive use of deadly force policies and supervisor oversight [27, 45, 51, 53, 57]. Our finding bolsters the notion that personnel incentives may be one agency policy that can mitigate deadly force, particularly against people of color.

### Video use

Opposite our hypothesis, our finding that video use was associated with higher White civilian rates was likewise novel. Most studies, including randomized control trials, solely examine the influence of body cameras on officer force, and findings are mixed [59, 67, 76, 77, 100–103]. To compare findings to this literature, we evaluated body camera use independently (vs. 4 video types) and the association was also positive. These combined results underscore our lack of understanding about how video use truly impacts officer behavior, meriting continued study.

### Marginal findings

A marginally significant association suggests that additional training for new-hires trends toward higher rates of all civilian killings, which aligns with previous work showing a positive association between officer field training and police killings [58]. The type of additional new officer training (e.g., racial bias, de-escalation vs. physical restraint) may be reflective of internal agency dynamics, which has a strong influence on officer use of lethal force [48, 104]. Therefore, more officer training is not likely to change officer behavior unless the agency culture and type of training demands it. In LEMAS, the additional training was not characterized, so there is no way to know what type of training(s) may have unintended fatal consequences, further indicating a cautious interpretation of this finding.

External statistical analysis and less-lethal weapons use were marginally associated with Black civilian killings. Having an external organization conduct internal research trended toward lower rates. Analysis type was not reported (e.g., crime, geospatial), thus the specific research that may be protective is unknown. Plausibly, agencies that allow external organizations to examine internal performance may promote wider transparency and greater agency liability, which in turn, contributes to less use of officer force, particularly against Black civilians, thereby reducing police killings and perhaps racial inequities.

Conversely, less-lethal weapons use was positively associated with a marginally higher Black civilian rate. However, most publications report on the impact of conductive energy devices (CED) (e.g., Tasers) on police use of deadly force. The overall CED literature points to (1) greater officer use of CED than firearms when confronting potentially lethal suspect resistance [105], (2) reduced civilian and officer injuries when used [62], and (3) more restrictive policies reducing both CED use [51] and use of force overall [27]. Therefore, we assessed Taser use alone (vs. our composite measure) and our results were null, which aligns with other literature [98, 106]. Our finding supports public concern that alternate less-lethal weapon use may leave civilians of color at higher risk (e.g., neck restraints). Future studies should investigate the independent impacts of various non-lethal tactics on police killing rates among racially minoritized groups.

The absence of any statistically significant policy associations among agencies involved in Black killings is an important finding. One interpretation is that our models lacked sufficient statistical power to detect an association due to a modest number of agencies involved in Black killings (*N = 165*). Another interpretation is that the policies we examined are ineffective at reducing the killings of Black people. This includes policies that specifically purport to protect Black lives, such as the use of body cameras and less-lethal weapons training. This finding has urgent policy implications. Though support for defunding or reforming police departments is increasing, past reform efforts have not reduced police violence or improved community health [107]. For example, officer body camera use has increased since the 2014 killing of Michael Brown yet police shootings largely remain unchanged [108]. Two recent policy statements passed by the American Public Health Association identify law enforcement and the carceral system more broadly as a threat to the public’s health, calling for a disinvestment in and abolition of these systems and investment in alternative strategies to promote equitable community health and wellbeing [109, 110]. Critical to this recommendation is the stance that additional reforms of an inherently violent and racist social institution will fail. Our finding that a host of different policy reforms are ineffective at protecting Black lives supports this conclusion.

### Limitations and strengths

Our exploratory study findings should be interpreted with caution. Causal inference cannot be inferred using this cross-sectional design. Though temporality was established as each policy was implemented prior to each fatality, LEMAS does not track or report the ongoing active status of agency policies, hence it is unclear whether policies remained implemented at time of death. Several factors limit generalizability. US law enforcement agencies were not required to participate; however, LEMAS surveyed a random sample and the response rate was high (85%) thus the agencies included should be fairly representative. External validity is further compromised by half of cases being excluded. Despite the high response rate, there may be some self-selection bias introduced in terms of organizational or cultural differences between agencies that opted in and out of participation. Apart from race, we found no differences in matched and unmatched cases (S1 Table). However, differences between the matched and unmatched agencies emerged, suggesting bias resulting from unobserved variation in agency characteristics (S2 Table). Because law enforcement agencies are not required to collect or report information associated with agency policies, police–civilian encounters, or use of force events to a national registry [70, 111–113], there is no way to test this assumption. Moreover, we are unable to predict the direction of the bias without knowing the distribution of the outcome in unmatched agencies during other years. Though not formally validated in association with deadly use of force events, several LEMAS measures appear consistent with other findings [17, 58]. Internal validity also largely depends on accurate data collection and proper validation by *The Guardian*. Although The Counted database is considered reliable [73, 74], it is improbable that all known cases are accurately reported to any system, conceivably leading to an underestimate of the true prevalence of police killings. Last, due to a small sample size, the likelihood of a committing a *Type II error* was higher for agency estimates involving race-specific civilian killings.

## Conclusion

Police use of fatal force is a public health crisis with countless adverse direct and indirect impacts on individuals and communities. US law enforcement agency policies may reduce avoidable police killings. However, policy predictors mitigating fatalities show variation by racial group with little to no benefit offered to Black civilians. Racial inequity in fatalities undermines police effectiveness and contributes to racial health disparities. Hence, impact evaluations and meta-analyses of law enforcement reform efforts to protect Black lives should be explored. To build upon the current research, a follow-up investigation among all LEMAS agencies, those with and without fatalities, would provide deeper insight into whether and to what extent each policy may predict the risk of civilian deaths caused by law enforcement nationwide. Importantly, better data for rigorous research is essential to address this issue. Primary prevention efforts focused on investing in communities, rather than policing, is critical for truly achieving community safety, equity, and wellbeing.

## Supporting information

S1 Fig Directed acyclic graph representing working knowledge of factors linking US law enforcement policies and police killings. (PNG)

S1 Table Sample distribution of civilian characteristics in The Counted by agency-match status (N = 2,238). (DOCX)

S2 Table Sample distribution of 2013 LEMAS agency policy by case-match status (N = 2,826). (DOCX)

S3 Table Bivariate analyses between covariates and 2013 LEMAS agency policies and police killings in The Counted during 2015–16 (N = 449). (DOCX)
