# Supplementary figures and images for: US law enforcement policy predictors of race-specific police fatalities during 2015–16
Source: PLoS One. 2021 Jun 23;16(6):e0252749. doi: 10.1371/journal.pone.0252749 (PMC8221500; doi:10.1371/journal.pone.0252749)

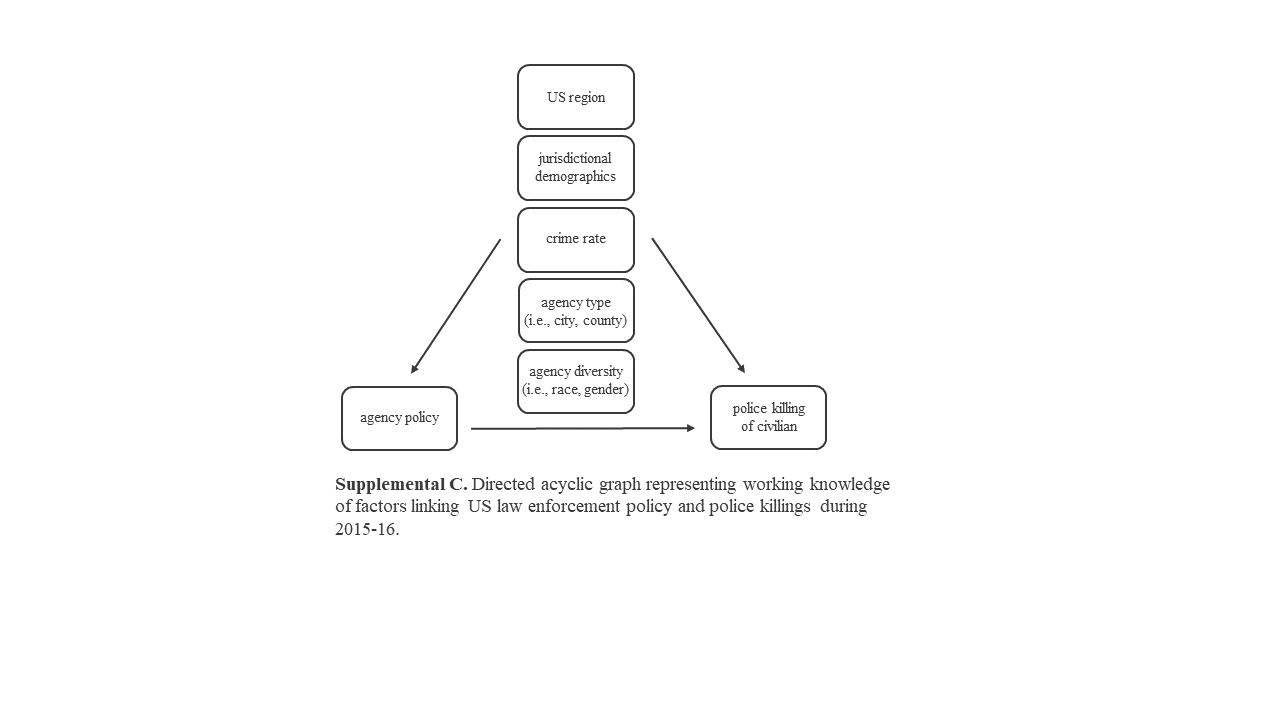

Supplement: S1 Fig — (PNG) [file pone.0252749.s001.png]
