# Supplementary material for: US law enforcement policy predictors of race-specific police fatalities during 2015–16
Source: PLoS One. 2021 Jun 23;16(6):e0252749. doi: 10.1371/journal.pone.0252749 (PMC8221500; doi:10.1371/journal.pone.0252749)
Supplement: S1 Table — (DOCX) [file pone.0252749.s002.docx]

| S1 Table. Sample distribution of civilian characteristics in The Counted by agency-match status *(N=2,238)* | | | | | | |  |
| --- | --- | --- | --- | --- | --- | --- | --- |
| **Characteristic** | **Matched** (*N=1,085*) | | **Unmatched** (*N=1,153*) | | P-value | |  |
|  | *n (%)* | | | |  | |  |
| Age | |  | |  | | 0.053 | |
| < 25 | 495 (46) | | 464 (40) | |  | |  |
| 25-34 | 247 (23) | | 296 (26) | |  | |  |
| 34-44 | 187 (17) | | 191 (17) | |  | |  |
| 45-54 | 102 (9) | | 130 (11) | |  | |  |
| > 55 | 54 (5) | | 72 (6) | |  | |  |
| Race/ethnicity |  | |  | | 0.000 | |  |
| White | 512 (47) | | 668 (58) | |  | |  |
| Black | 311 (29) | | 254 (22) | |  | |  |
| Hispanic/Latino | 206 (19) | | 160 (14) | |  | |  |
| Other/Unknown | 56 (5) | | 71 (6) | |  | |  |
| Gender |  | |  | | 0.820 | |  |
| Male | 54 (5) | | 55 (5) | |  | |  |
| Female | 1031 (95) | | 1098 (95) | |  | |  |
| Armed |  | |  | | 0.448 | |  |
| No | 188 (17) | | 214 (19) | |  | |  |
| Yes | 897 (83) | | 939 (81) | |  | |  |
| Firearm |  | |  | | 0.200 | |  |
| No | 492 (45) | | 554 (48) | |  | |  |
| Yes | 593 (55) | | 599 (52) | |  | |  |
| Killed by gunshot |  | |  | | 0.117 | |  |
| No | 91 (8) | | 119 (10) | |  | |  |
| Yes | 994 (92) | | 1034 (90) | |  | |  |
| *Note: Multiple agencies can be linked to any given killing. Thus, the total number of matched cases in The Counted (N=1,085) is less than the total cases in the analysis (N=1,211) because more than one agency was linked to 126 of the matched cases.* | | | | | | |  |
| *P-values: ANOVA F-test (continuous-categorical); Chi-square test (categorical-categorical).* | | | | | | |  |
