# Supplementary material for: US law enforcement policy predictors of race-specific police fatalities during 2015–16
Source: PLoS One. 2021 Jun 23;16(6):e0252749. doi: 10.1371/journal.pone.0252749 (PMC8221500; doi:10.1371/journal.pone.0252749)
Supplement: S2 Table — (DOCX) [file pone.0252749.s003.docx]

| S2 Table. Sample distribution of 2013 LEMAS agency policy by case-match status (*N=2,826*) | | | |
| --- | --- | --- | --- |
| **Policy/Practice** | **Matched** *(N=480)* | **Unmatched** *(N=2,328)* | P-value |
|  | *n (%)* | |  |
| % Full-time personnel |  |  | 0.000 |
| *mean (±SD)* | 95.16 (7.32) | 86.74 (18.72) |  |
| Collective bargaining offered |  |  | 0.498 |
| No | 81 (17) | 364 (16) |  |
| Yes | 399 (83) | 1964 (84) |  |
| ^Ɨ^Active collective bargaining |  |  | 0.000 |
| No | 172 (43) | 1073 (55) |  |
| Yes | 227 (57) | 891 (45) |  |
| Mission statement |  |  | 0.000 |
| No | 20 (4) | 360 (15) |  |
| Yes | 460 (96) | 1962 (85) |  |
| Minimum education required |  |  | 0.461 |
| None | 4 (1) | 30 (1) |  |
| HS or other | 391 (81) | 1926 (83) |  |
| Some college | 71 (15) | 326 (14) |  |
| Bachelor's degree | 14 (3) | 46 (2) |  |
| Additional training for new hires |  |  | 0.003 |
| No | 49 (10) | 358 (15) |  |
| Yes | 431 (90) | 1970 (85) |  |
| Community policing training |  |  | 0.173 |
| No | 166 (35) | 882 (38) |  |
| Yes | 314 (65) | 1446 (62) |  |
| Less-lethal weapons |  |  | 0.000 |
| *range 0-9; mean (±SD)* | 7.12 (1.54) | 6.16 (1.74) |  |
| Video use |  |  | 0.038 |
| *range 0-3; mean (±SD)* | 1.41 (0.87) | 1.32 (0.89) |  |
| Community evaluation |  |  | 0.000 |
| No | 271 (56) | 1692 (73) |  |
| Yes | 209 (44) | 636 (27) |  |
| Incentives |  |  | 0.000 |
| *range 0-9; mean (±SD)* | 3.15 (1.90) | 1.95 (1.70) |  |
| Any statistical analysis |  |  | 0.000 |
| No | 43 (9) | 821 (35) |  |
| Yes | 437 (91) | 1507 (65) |  |
| ^£^External statistical analysis |  |  | 0.000 |
| No | 331 (69) | 1188 (79) |  |
| Yes | 106 (31) | 319 (21) |  |
| % Female personnel |  |  | 0.000 |
| *mean (±SD)* | 10.7 (5.6) | 8.90 (8.72) |  |
| % Full-time White personnel |  |  | 0.000 |
| *mean (±SD)* | 75.0 (24.6) | 83.79 (23.71) |  |
| *Abbreviations: LEMAS=Law Enforcement Management and Administrative Statistics; HS=high school, SD=standard deviation.* | | | |
| *Note: 18 cases dropped due to mismatches between agency name and state.* | | | |
| *P-values: ANOVA F-test (continuous-categorical); Chi-square test (categorical-categorical).* | | | |
| ^Ɨ^*Matched n=399, unmatched n=1964.* | | | |
| ^£^*All Matched n=437, unmatched n=1507.* | | | |
