# Supplementary material for: US law enforcement policy predictors of race-specific police fatalities during 2015–16
Source: PLoS One. 2021 Jun 23;16(6):e0252749. doi: 10.1371/journal.pone.0252749 (PMC8221500; doi:10.1371/journal.pone.0252749)
Supplement: Supplementary file 4 [file pone.0252749.s004.docx]

Supplemental D. Bivariate analysis between covariates and 2013 LEMAS agency policies and police killings in The Counted during 2015-16 (*N=449*)

	Agency type	Number of officers		% Female personnel	% Full-time White personnel	Median age	% US native	% HS Diploma	% BA/BS Degree	Gini index	Median income (K)	% Below poverty	% Married	% Owner occupied housing units	Violent crime rate (per 100K)	% Black population	Total population size
Exposure	P-value or Pearson correlation coefficient																
% Full-time personnel	0.218	0.000	0.208	*r=0.050*	*r=-0.071*	***r=-0.116***	***r=-0.132***	*r=0.001*	***r=0.119***	*r=0.019*	*r=0.051*	*r=-0.003*	*r=-0.086*	***r=-0.109***	***r=0.148***	*r=0.070*	***r=0.103***
Collective bargaining offered	0.001	0.610	0.000	0.627	0.084	0.381	0.000	0.284	0.960	0.840	0.147	0.544	0.523	0.054	0.157	0.511	0.146
Active collective bargaining *(n=374)*	0.000	0.281	0.000	0.116	0.856	0.122	0.001	0.567	0.453	0.213	0.131	0.969	0.059	0.015	0.243	0.084	0.524
Mission statement	0.108	0.000	0.223	0.458	0.408	0.103	0.016	0.381	0.018	0.812	0.113	0.429	0.241	0.003	0.033	0.223	0.278
Minimum education required	0.001	0.083	0.513	0.020	0.786	0.078	0.032	0.086	0.000	0.871	0.003	0.088	0.851	0.128	0.377	0.046	0.068
Additional training for new hires	0.719	0.240	0.355	0.580	0.831	0.888	0.716	0.062	0.036	0.503	0.434	0.267	0.722	0.168	0.927	0.263	0.231
Community policing training	0.466	0.262	0.847	0.693	0.795	0.515	0.943	0.275	0.342	0.462	0.349	0.874	0.521	0.351	0.636	0.032	0.142
Less-lethal weapons	0.782	0.003	0.000	*r=0.033*	*r=0.002*	*r=-0.032*	***r=-0.156***	*r=0.070*	*r=0.144*	*r=-0.049*	***r=0.180***	***r=-0.167***	*r=0.075*	*r=-0.004*	*r=-0.079*	*r=-0.049*	*r=0.072*
Video use	0.827	0.800	0.000	*r=0.063*	*r=0.088*	*r=0.043*	***r=0.254***	***r=0.099***	*r=-0.018*	*r=-0.010*	***r=-0.118***	*r=-0.022*	*r=-0.021*	*r=0.088*	*r=-0.030*	*r=0.059*	*r=0.471*
Community evaluation	0.166	0.000	0.535	0.000	0.611	0.842	0.029	0.202	0.000	0.733	0.065	0.154	0.128	0.021	0.160	0.031	0.003
Incentives	0.004	0.000	0.000	*r=0.073*	***r=-0.201***	*r=-0.090*	***r=-0.376***	*r=-0.154*	***r=0.103***	*r=0.062*	***r=0.190***	*r=-0.059*	*r=-0.054*	***r=-0.198***	***r=0.107***	***r=0.116***	***r=0.237***
Any statistical analysis	0.000	0.000	0.516	0.059	0.086	0.007	0.000	0.922	0.032	0.547	0.166	0.824	0.003	0.000	0.000	0.082	0.069
External statistical analysis *(n=409)*	0.103	0.053	0.093	0.007	0.357	0.087	0.704	0.957	0.372	0.898	0.564	0.299	0.004	0.247	0.018	0.000	0.001
Outcome																	
Agencies w/Killings (N=*449*)	0.007	0.000	0.050	***r=0.177***	***r=-0.186***	***r=-0.129***	***r=-0.213***	***r=-0.128***	***r=0.086***	***r=0.098***	*r=0.008*	*r=-0.063*	***r=-0.153***	***r=-0.237***	***r=0.212***	***r=0.425***	***r=0.595***
Agencies w/White Killings (*n=297*)	0.412	0.001	0.051	*r=0.091*	*r=-0.091*	*r=-0.089*	***r=-0.153***	***r=-0.153***	*r=-0.074*	*r=0.048*	*r=-0.001*	*r=-0.047*	***r=--0.131***	***r=--0.173***	***r=0.150***	***r=0.160***	***r=0.414***
Agencies w/Black Killings *(n=165)*	0.177	0.034	0.415	***r=0.248***	***r=-0.175***	*r=-0.148*	*r=-0.103*	*r=-0.082*	*r=0.102*	*r=0.079*	*r=-0.014*	*r=0.054*	***r=-0.165***	***r=-0.215***	***r=0.257***	***r=0.631***	***r=0.479***
Agencies w/non-White Killings	0.014	0.002	0.346	***r=0.176***	***r=-0.172***	*r=-0.115*	***r=-0.210***	***r=-0.132***	*r=0.086*	*r=0.070*	*r=0.040*	*r=0.031*	*r=-0.120*	***r=-0.241***	***r=0.180***	***r=0.469***	***r=0.614***

r=Pearson correlation coefficient (continuous-continuous variables); ANOVA F-test (continuous-categorical variables); Chi-square test (categorical-categorical variables); ***Bolded*** *=P<0.05*
